# Extensive Genetic Connectivity and Historical Persistence Are Features of Two Widespread Tree Species in the Ancient Pilbara Region of Western Australia

**DOI:** 10.3390/genes11080863

**Published:** 2020-07-29

**Authors:** Heidi M. Nistelberger, Rachel M. Binks, Stephen van Leeuwen, David J. Coates, Shelley L. McArthur, Bronwyn M. Macdonald, Margaret Hankinson, Margaret Byrne

**Affiliations:** Department of Biodiversity, Conservation and Attractions, Biodiversity and Conservation Science, Locked Bag 104, Bentley Delivery Centre, Perth, WA 6983, Australia; rachel.binks@dbca.wa.gov.au (R.M.B.); sleeuwen@amnet.net.au (S.v.L.); dave.coates@dbca.wa.gov.au (D.J.C.); shelley.mcarthur@dbca.wa.gov.au (S.L.M.); Bronwyn.Macdonald@dbca.wa.gov.au (B.M.M.); Margaret.Hankinson@dbca.wa.gov.au (M.H.); Margaret.Byrne@dbca.wa.gov.au (M.B.)

**Keywords:** *Acacia*, *Corymbia*, genetic diversity, phylogeography, dispersal, seed dispersal

## Abstract

Phylogeographic studies can be used as a tool to understand the evolutionary history of a landscape, including the major drivers of species distributions and diversity. Extensive research has been conducted on phylogeographic patterns of species found in northern hemisphere landscapes that were affected by glaciations, yet the body of literature for older, unaffected landscapes is still underrepresented. The Pilbara region of north-western Australia is an ancient and vast landscape that is topographically complex, consisting of plateaus, gorges, valleys, and ranges, and experiences extreme meteorological phenomena including seasonal cyclonic activity. These features are expected to influence patterns of genetic structuring throughout the landscape either by promoting or restricting the movement of pollen and seed. Whilst a growing body of literature exists for the fauna endemic to this region, less is known about the forces shaping the evolution of plant taxa. In this study we investigate the phylogeography of two iconic Pilbara tree species, the Hamersley Bloodwood (*Corymbia hamersleyana*) and Western Gidgee (*Acacia pruinocarpa*), by assessing patterns of variation and structure in several chloroplast DNA regions and nuclear microsatellite loci developed for each species. Gene flow was found to be extensive in both taxa and there was evidence of long-distance seed dispersal across the region (pollen to seed ratios of 6.67 and 2.96 for *C. hamersleyana* and *A. pruinocarpa*, respectively), which may result from flooding and strong wind gusts associated with extreme cyclonic activity. Both species possessed high levels of cpDNA genetic diversity in comparison to those from formerly glaciated landscapes (*C. hamersleyana* = 14 haplotypes, *A. pruinocarpa* = 37 haplotypes) and showed evidence of deep lineage diversification occurring from the late Miocene, a time of intensifying aridity in this landscape that appears to be a critical driver of evolution in Pilbara taxa. In contrast to another study, we did not find evidence for topographic features acting as refugia for the widely sampled *C. hamersleyana*.

## 1. Introduction

Studies of intraspecific phylogeography provide insight into the evolutionary history of a species, revealing forces that have shaped its distribution and diversity. When phylogeographic data for multiple co-occurring species are available, we can infer the evolutionary history of a landscape, identifying key climatic phenomena, landscape features and forces that have collectively shaped species’ abundance and distribution. Comparative phylogeography, for example, revealed the varied responses of species to glaciation during the last glacial maximum (LGM), including the consequences of expansion from major refugia [1] as well as persistence in localised idiosyncratic refugia within major refugial regions [2,3]. Comparative phylogeographic studies of old landscapes that have remained unglaciated, including the Greater Cape Floristic Region (GCFR) of southern Africa, the Californian Floristic Province (CA-FP), the South Western Australian Floristic Region (SWAFR), the Iberian Peninsula, and parts of eastern North America, have identified high genetic diversity and structure in comparison to younger landscapes affected by glaciations [2,4,5,6,7,8]. This has been attributed both to a lack of extinction due to relative climate stability and extensive localised persistence of populations allowing for the accrual of genetic variation [9,10]. In addition, areas of topographic complexity and/or climatically buffered regions within older landscapes have also been identified as putative refugia for many species during periods of climate fluctuation, yet these patterns are often idiosyncratic in nature, with locations of refugia varying amongst taxa [7,11,12,13,14]. For example, studies in the SWAFR have highlighted the species-specific responses to the changing climate, with many showing long-term persistence in localised refugia and no signal of major contraction or expansion [7,15]. Alternatively, some species show persistence in major refugia with signals of expansion [11,13] and others show persistence across large areas with localised refugia and signals of historical expansion as aridification progressed [14,16,17,18,19]. Thus, drawing broad conclusions from these older landscapes is hampered by the long history of species’ persistence as well as the individualised responses of species and populations to landscape features and climate phenomena [8].

The Pilbara region, in the north of Western Australia is another vast and ancient landscape, yet until recently [20,21,22] there have been few studies investigating intraspecific phylogeography and the evolutionary history of plant species across this region. It is topographically complex, composed of undulating hills, rocky outcrops, and alluvial plains that reflect ancient and contemporary drainage systems (reviewed in [23]). It is one of the oldest landmasses on earth and has been free of glacial ice since the Permo-Carboniferous glaciation (280–320 Ma) [24]. Similar to other ancient landscapes [25,26,27], it is a region of high species diversity and endemism with approximately 1800 plant species recognised, 15% of which are endemic [28]. The Pilbara is subject to extreme meteorological phenomena and is one of the most cyclone-prone regions in the world [29]. The strong winds and flooding associated with seasonal cyclonic activity and tropical depressions have been hypothesised to facilitate gene flow amongst plants [20,21]. This cyclonic activity, combined with the complex topography and lack of glaciation, is anticipated to have driven complex evolutionary patterns in species endemic to this region. Analyses of faunal assemblages are so far consistent with these expectations and show idiosyncratic patterns of species phylogeography that reflect the influence of regional habitat differences and vicariant events as well as stochastic population processes on the development of genetic diversity and structure [23]. Similar to other unglaciated landscapes, we predict plant species in the Pilbara to be characterised by higher levels of genetic diversity owing to historical persistence. However, the presence of genetic structure will be dependent upon the degree of historical and contemporary gene flow.

**Figure 1:**
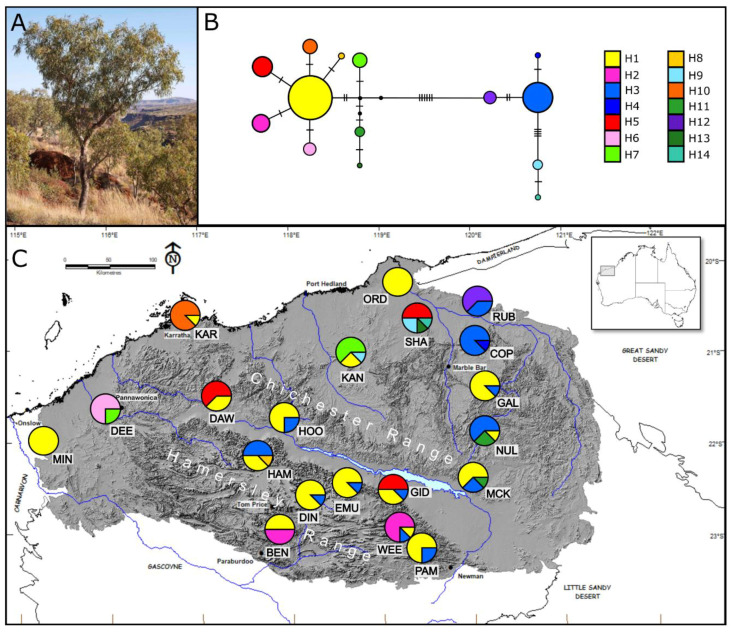
(**A**) *C. hamersleyana* at East Munjina (EMU); photo credit S. van Leeuwen. (**B**) Network of cpDNA haplotypes identified with frequency indicated by circle size and black dashes representing mutational steps. (**C**) Map of the 20 *C. hamersleyana* populations with pie charts indicating frequency of haplotypes at each population.

**Figure 2:**
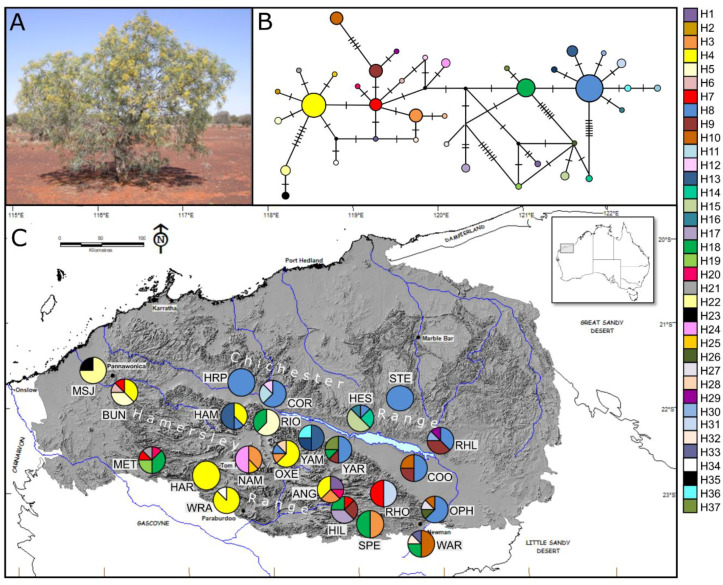
(**A**) *A. pruinocarpa* at Warrawanda (WAR); photo credit S. van Leeuwen. (**B**) Network of cpDNA haplotypes identified with frequency indicated by circle size and black dashes representing mutational steps. (**C**) Map of the 23 *A. pruinocarpa* populations with pie charts indicating frequency of haplotypes at each population.

Phylogeographic studies of two widespread shrub species from the Pilbara showed high genetic diversity but a lack of phylogeographic structure [20,21]. This is remarkable given the vast distances and complex environments inhabited by these taxa. The lack of structure has been attributed to extensive pollen flow as well as opportunities for seed dispersal, the latter likely facilitated by strong wind gusts and localised flooding throughout the landscape [20,21]. One of the studies identified the inland Hamersley and Chichester ranges as putative historical refugia based on higher genetic diversity associated with those features (see Figure 1 and Figure 2) and a signal of expansion in the surrounding lowlands [21]. Both studies [20,21] indicate that topographic complexity and climatic factors may be important drivers of genetic variation in Pilbara plants. 

To assess whether the patterns observed in widespread Pilbara flora are idiosyncratic or reflective of broader patterns throughout the landscape, we expand upon the body of work by examining patterns of phylogeography in two iconic trees, the Hamersley Bloodwood (*C. hamersleyana*) and Western Gidgee (*A. pruinocarpa*). Both species are common and widespread, forming conspicuous components of the floristic community. They have distributions across a range of features, including rocky outcrops and ranges, as well as along alluvial plains and drainage lines, which provides an ideal opportunity to explore drivers of evolution in this landscape. Using a combination of nuclear microsatellite markers and chloroplast DNA sequences we aimed to determine whether: (a) Tree species show evidence of historical persistence in the landscape through a high haplotype number and diversity; (b) there is higher genetic diversity associated with topographic features such as the Hamersley and Chichester Ranges, and lower diversity in surrounding areas as evidence for these features acting as historical refugia for the widely sampled *C. hamersleyana*; and (c) there is evidence of genetic connectivity across the landscape, resulting in a lack of genetic structure as previously observed in two other widespread Pilbara tree species [20,21].

## 2. Materials and Methods

### 2.1. Study Species

*C. hamersleyana* ((D.J. Carr and S.G.M. Carr) K.D. Hill and L.A.S. Johnson), commonly known as the Hamersley Bloodwood, is widely distributed throughout the Western Australian Pilbara region (Figure 1). It grows up to 12 m in height and can occur in tree or mallee form on rocky sites and hilltops as well as on shallow sands in depressions and lowlands. The flowering period is extensive, lasting from April to September [30] and the species is likely to be pollinated by both birds and insects. Similar to other desert bloodwoods, the seed is ellipsoid in shape and possesses a terminal wing that may assist with dispersal. The species was previously described as two taxa, *C. hamersleyana* and *C. semiclara*, based on differences in the scurfiness of buds, the length of pedicels and glossiness of leaves, and recognition by Aboriginal people in the region of differences between trees occupying the desert plains and flats along creeks and those occurring on stony crests [30]. However, the two taxa were later synonymised due to the difficulty in assessing diagnostic characters that were often found to overlap. Populations of intermediate trees suspected to result from ancestral interbreeding were recorded to occur in the north and east of the Hamersley Range. It was also noted that there was an apparent intergrade into another bloodwood *C. opaca* (D.J. Carr and S.G.M. Carr) in the east. These gradations suggest that hybridisation may be an important feature of the Hamersley Bloodwood [30]. 

*A. pruinocarpa* (Tindale), commonly known as the Western Gidgee or Burlurru [31], occurs as a 3–12 m high shrub or tree and is found on sandy, loamy or stony soils in arid regions from the Pilbara to the inland Murchison in Western Australia (Figure 2), and extending eastwards as far as north-western South Australia and central-western Northern Territory [32]. It occurs on both upland regions including the Chichester and Hamersley Ranges and in lower lying regions to the south of the Hamersley Ranges. Due to sampling restrictions only populations occurring in the Pilbara region were sampled as part of this study. *A. pruinocarpa* produces a profusion of large golden flowers from October to December and is capable of clonal growth via root-suckering. Insects are likely to be the dominant pollinators. The seeds are small, flat, and black with a small aril, which suggests ants are typical seed dispersers [32]. 

### 2.2. Sampling 

Leaf material was collected from 24 non-adjacent individuals from 20 populations of *C. hamersleyana* (*n* = 480) and 23 populations of *A. pruinocarpa* (*n* = 460) across the Pilbara region of Western Australia (Figure 1 and Figure 2) (Tables S1 and S2). Following freeze drying, genomic DNA was extracted using a 2% CTAB method [33], modified by adding 1% polyvinylpyrrolodine to the extraction buffer. All 24 individuals per population were genotyped using 14 species-specific microsatellite markers developed via partial genome sequencing on a 454 platform at the Australian Genome Research Facility (AGRF, Perth) (see Table S3 for primer details). A subset of eight randomly selected individuals from each population were sequenced at three (*C. hamersleyana*) and four (*A. pruinocarpa*) chloroplast sequence regions known to display intraspecific variation [34].

### 2.3. Microsatellite Data

Microsatellite development was based on the methodology of Gardner et al., (2011) [35]. Following shotgun sequencing, QDD v.2.0 [36] was used to identify unique microsatellite loci and design primers for 600 potential loci for each of *C. hamersleyana* and *A. pruinocarpa*. For each species, preliminary tests were conducted on 40 loci using a subset of individuals to assess amplification success, variability, and scoring consistency. This process resulted in 14 informative microsatellite loci for each species that showed clear, reproducible banding patterns (Table S3). Microsatellite amplification was performed in 7.25 µL multiplexed reactions containing 3.5 µL Qiagen Multiplex PCR Master Mix (Vic, Australia), 0.75 µL primer mix, 2 µL sterile distilled water, and 1 µL (10 ng) of template DNA. Cycling conditions were the same for all loci for both species; 95 °C for 15 min, followed by 35 cycles of 94 °C for 30 s, 60 °C for 90 s, and 72 °C for 60 s, followed by a final extension of 60 °C for 30 min (Eppendorf Mastercycler). PCR products were electrophoresed on an Applied Biosystems 3730 Sequencer (Murdoch University) and genotypes scored using GENEMAPPER™ v.3.7 (Applied Biosystems). Given *A. pruinocarpa* is capable of clonal reproduction, any individuals displaying identical multi-locus genotypes were pruned so that the final data set contained genets only. 

### 2.4. Microsatellite Analysis

The 14 loci for each species were assessed for genotypic disequilibrium in GENEPOP v.4.2 [37] with significance levels adjusted for multiple testing with sequential Bonferroni correction [38]. FREENA [39] was used to estimate potential null allele frequencies following the expectation maximization (EM) algorithm [40]. Estimates of genetic diversity, including percentage polymorphism, allelic richness, the number of private alleles, and observed and expected heterozygosity were estimated in GENALEX v.6.1 [41]. The fixation index of individuals relative to subpopulations (*F*_IS_) was estimated in GENEPOP and significance values determined with 10,000 exact tests.

GENALEX was used to assess genetic differentiation as *F*_ST_ [42] (a) among individuals (visualised with principal coordinates analysis: PCoA) and (b) among populations (visualised as a heatmap using the ‘pheatmap’ package in R [43]. The differentiation estimate *R*_ST_ [44] which takes into account the strict stepwise mutational process occurring at microsatellite loci was also estimated in SPAGEDI [45]. Structuring of genetic diversity was further explored without prior information on geographic origin and population membership by means of the Bayesian approach implemented in STRUCTURE v.2.3.4 [46]. We selected the admixture ancestry model with correlated allele frequencies given the likelihood of high population similarity due to the gene flow [47]. Clustering analyses were run for *K* = 1–20 with 10 replicates for each *K*, a burning of 100,000 generations and 500,000 further Monte Carlo Markov chain (MCMC) generations. Run consistency was assessed with CLUMMP v.1.1.2 [48] and the value of *K* with the highest posterior probability was determined with STRUCTURE HARVESTER v.0.6.93 [49]. The distribution of alleles across the sampled region was assessed with a Mantel test (9999 permutations) for isolation by distance, using transformed pairwise *F*_ST_ values (*F*_ST_/(1 − *F*_ST_) for genetic distance and the natural logarithm of geographical distance.

### 2.5. Chloroplast DNA (cpDNA) Amplification and Sequencing

Three chloroplast DNA regions were sequenced for *C. hamersleyana*: The ribosomal protein rpL16 intron, the intergenic spacer ndhC-trnV, and the tRNA-Gly (trnG) intron. For *A. pruinocarpa*, samples were sequenced at the intergenic spacers ndhF-rpl32, rpl32-trnL, trnS-trnG, and psbD-trnT. Amplifications were carried out according to the protocols listed in [34] with thermocycling conditions according to [50]. A Serapure method was used to purify PCR products [51] which were then sequenced at the Australian Genome Research Facility (Perth). Sequence data were aligned using CLUSTAL W [52] and corrected by the eye where necessary using BIOEDIT [53]. Indels arising from mononucleotide repeats were removed as these are often introduced via an error during the PCR process and are difficult to accurately score [54].

### 2.6. cpDNA Analysis

The number of haplotypes and haplotype diversity including indels were determined using DnaSP v.6 [55]. Subsequent analyses were conducted on data in which indels were manually encoded as transitions. DnaSP was also used to calculate three measures of neutrality that also predict population size changes under Wright Fisher assumptions of panmixia and constant population size: Tajima’s D [56], Fu’s *F*_s_ [57], and Ramos Onsins and Rozas *R*_2_. The latter statistic compares the difference between the number of singleton mutations and the average number of nucleotide differences and has greater power than Tajima’s D and Fu’s *F*_S_ when testing smaller datasets [58]. For all three estimates, tests of significance were estimated by comparing the observed values with distributions generated with 10,000 replicates of coalescent simulations under the standard neutral model. Analysis of molecular variance (AMOVA) was performed using Arlequin v.3.5.2.2 [59] along with tests of demographic and spatial expansion using Goodness of Fit tests (Harpending’s raggedness index (Hrag)) and the Sum of Squared Deviations (SSD) to determine significance. To investigate the presence of phylogeographic structure, where closely related haplotypes group together in space, we tested whether the measure of genetic differentiation *N*_ST_, which considers not just differences in haplotype frequencies but also genetic distances, was significantly higher than *G*_ST_ using the U test in the program PERMUT v.2.0 [60]. Networks of haplotype relationships were generated in NETWORK v.5.0.1.1 (fluxus-engineering.com) using the Median-Joining method [61] and the Maximum Parsimony (MP) processing option [62]. The relative influence of pollen and seed gene flow were estimated using Equation (5a) from Ennos, 1994 [63]. 

Evolutionary relationships were investigated using Bayesian analysis in BEAST v.1.10 [64]. For *C. hamersleyana*, the south-western Australian endemic *Eucalyptus marginata* (Donn ex Sm) (GenBank acc. KC180781) and two *Corymbia* species, *C. citriodora* ((Hook.) K.D. Hill and L.A.S. Johnson) (GenBank acc. KP015029) and *C. tesselaris* ((*F.Muell.*) K.D. Hill and L.A.S. Johnson) (GenBank acc. KC180803) were used as outgroups. *C. citriodora* and *C. tesselaris* possess overlapping distributions that are centred throughout Queensland and northern New South Wales. Bayesian trees were dated according to two different methods and three different calibration dates. The first method (a) utilised the estimated stem age of the *Corymbia* (and *Angophora*) clade as 52–57 Ma [65] with a normal, mean distribution set around the root of 54 Ma and a standard deviation of 2.5 Ma. A calibrated YuleTree Prior [66] was selected along with a strict clock model owing to the relatively limited variation seen in the intraspecific dataset. The second method utilised two mean plastid DNA substitution rates for the *Angophora*/*Corymbia* group reported in Thornhill et al. (2012) [67], the first (b) based on macrofossil calibrations (7 × 10^−4^ s/s/Ma) and the second, (c) using combined root, pollen, and macrofossil calibrations (8.3 × 10^−4^ s/s/Ma) [67] under a Yule speciation prior [68]. For these analyses, a lognormal prior was applied to the strict clock rates with the standard deviations set to 1.0 × 10^−4^. For all analyses, a GTR+ invariant sites model of sequence evolution was implemented as identified using the corrected Akaike information criterion (AIC) in JModelTest2 [69]. 

Evolutionary relationships within *A. pruinocarpa* were assessed using the south-western Australian endemic *A. daphnifolia* (Meisn.) (GenBank acc. LN885259) as an outgroup. Highest credibility trees were dated according to the cpDNA substitution rate identified for the Mimosoid clade in [70] (4.83 × 10^−4^ s/s/Ma). This analysis utilised a lognormal prior around the strict clock rate with a standard deviation of 1.0 × 10^−4^ and a Yule speciation tree prior. An F81 + gamma model of sequence evolution was applied as determined by the corrected AIC in JModelTest2. 

For both species, final analyses involved combining four independent runs of 10 million generations. Convergence of each run was assessed using ESS values in TRACER v.1.7.1 [71]. The first 25% of combined trees were discarded as burning and the resulting Maximum Clade Credibility (MCC) trees were visualised using FigTree v.1.44 (http://tree.bio.ed.ac.uk/software/figtree/).

## 3. Results

### 3.1. Microsatellite Data

All microsatellite markers used in the study (Table S3) were polymorphic and showed independent inheritance with no indication of genotypic disequilibrium following Bonferroni correction (α′ 0.004). There was no indication of null alleles in either data set with no significant difference between estimates of *F*_ST_ based on ENA corrected and uncorrected allele frequencies (Table S4).

#### 3.1.1. *Corymbia hamersleyana*

**Figure 3:**
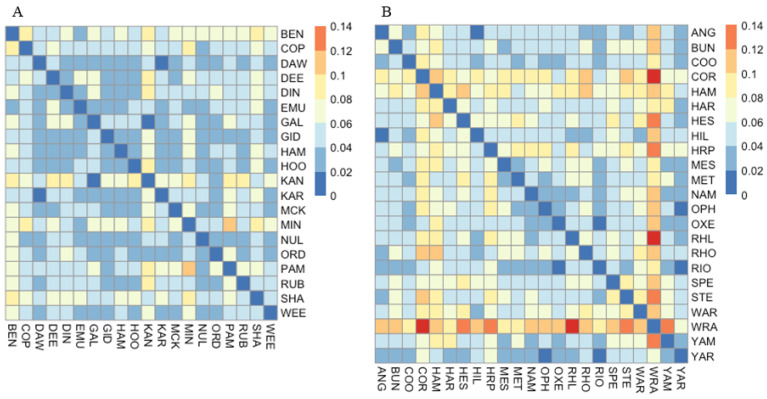
Heatmaps displaying pairwise population *F*_ST_ values (see legend) based on nuclear microsatellite data for (**A**) *C. hamersleyana* and (**B**) *A. pruinocarpa.*

**Figure 4:**
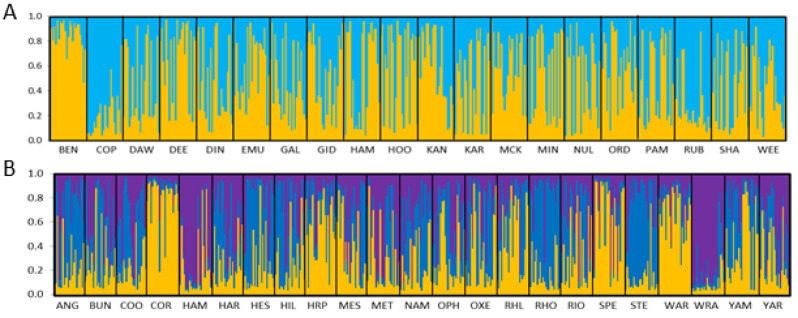
STRUCTURE analysis based on nuclear microsatellite data for (**A**) *C. hamersleyana* (*K* = 2) and (**B**) *A. pruinocarpa* (*K* = 3). Samples are ordered by population and each vertical bar represents an individual, with colours representing the proportion of assignment to each of the *K* genetic clusters.

Populations of *C. hamersleyana* showed moderate levels of genetic variation with expected heterozygosity ranging from 0.57 in HOO to 0.64 in DIN, SHA, and WEE (Table 1). There was no significant difference between the expected heterozygosity of populations occurring on the Chichester and Hamersley Ranges with those occurring on the surrounding lower lying regions (*t* = 0.15, *p* = 0.44). The average observed heterozygosity was 0.48 ± 0.01. Private alleles occurred in 12 of the 20 populations (Table 1). The fixation index was positive and highly significant in all populations with a mean *F*_IS_ of 0.19 ± 0.01 (Table 1). Estimates of pairwise population genetic differentiation were low across all populations (Figure 3) with an average pairwise *F*_ST_ of 0.05. *R*_ST_ estimates were also weak, with a global standardised estimate of 0.07. There was a weak but significant signal of isolation by distance with an *R* value of 0.293 (*p* = 0.003). Bayesian clustering analysis did not identify any meaningful genetic clusters with *K* = 2 identified (Figure 4A, Figure S1), and PCoA showed complete overlap of individuals across all populations (Figure S3). 

#### 3.1.2. *Acacia pruinocarpa*

A total of 18 individuals across 11 populations were identified as clonal ramets and were removed from the analysis, with final population sample sizes (20–24 individuals) listed in Table 1. Moderate levels of diversity were observed in the 14 microsatellite loci developed for *A. pruinocarpa* with expected heterozygosity ranging from 0.46 in RHO to 0.58 in BUN. Observed heterozygosity was lowest in STE (0.35) and highest in HAM (0.54) (Table 1). Private alleles were observed in 13 of the 23 populations. The fixation index (mean *F*_IS_ = 0.17 ± 0.01) indicated a significant deficit of heterozygotes in all populations except for the HAM population (Table 1). Genetic differentiation (*F*_ST_) was low across all populations (Figure 3) with a mean pairwise *F*_ST_ of 0.062 and mean *R*_ST_ of 0.06, and there was no evidence of isolation by distance across all individuals (*R* = 0.033, *p* = 0.367). The STRUCTURE analysis did not identify any strong patterns of genetic structure within *A. pruinocarpa*. The analysis resulted in three genetic clusters; however, most individuals were assigned to all three clusters in varying proportions (Figure 4b, Figure S2), a result also reflected in the PCoA, indicating that individuals represent one panmictic population (Figure S3).

### 3.2. cpDNA Data 

#### 3.2.1. *Corymbia hamersleyana*

The three cpDNA regions were concatenated to form a total length of 1588 bp consisting of six transitions, eight transversions, and five varying length indels. Fourteen unique haplotypes were identified with high frequency of the most common haplotype (43.13%, Hap 1) to low frequency of rare haplotypes (0.63%, Hap 4, 8, 13, and 14) (Figure 1). All populations displayed multiple haplotypes except for the peripheral populations MIN and ORD that possessed the common Hap 1. Half of the haplotypes (Hap 4, 6, 8, 10, 12, 13, and 14) were only identified in a single population (Figure 1) and most genetic variation was maintained within populations (61.6%) rather than among populations (38.4%). Haplotype diversity estimates (Table S1) in populations located on the Chichester and Hamersley Ranges did not differ significantly from populations found off the Ranges (*t* = −0.96, *p* = 0.349). There was no evidence of departure from neutrality in tests of Tajima’s D or Fu’s *F*_s_ (Table 2). Models of demographic and spatial expansions based on tests of the number of pairwise differences could not be rejected using both the Sum of Squares Deviation between the observed and expected mismatches and Goodness of fit tests based on Harpending’s raggedness index. Ramos Onsins and Rozas’ *R*_2_ statistic also supported a model of population growth (Table 2). The ratio of pollen to seed mediated gene flow was low at 6.67 95% CI [6.45–6.89] (Table 2).

#### 3.2.2. *Acacia pruniocarpa*

The four cpDNA regions were concatenated to a total of 2109 bp comprised of 12 transitions, 16 transversions, 10 varying length indels, and one multistate substitution. A total of 37 unique haplotypes were identified from 184 individuals (Table S2), ranging in frequency from 0.54% (Hap 2, 6, 12, 16, 20, 21, 25, 26, 27, 28, 29, 30, 32, 33, and 34) to 21.74% for the common Hap 8 (Figure 2). All populations possessed multiple haplotypes except STE and HRP where individuals displayed the most common haplotype (Hap 8), and HAR that possessed another common haplotype (Hap 4) (Figure 2). The majority (*n* = 29) of haplotypes were population specific with only eight haplotypes (Hap 3, 4, 7, 8, 9, 10, 13, and 18) shared amongst populations. AMOVA showed that 51.14% of the variation was partitioned within populations and 48.86% amongst populations. Tests of neutrality (Tajima’s D and Fu’s Fs) were non-significant but Ramos Onsin and Rozas’ *R*_2_ statistic was significant relative to expectations under the standard neutral model of evolution (Table 2). In addition, models of demographic and spatial expansion could not be rejected using tests of pairwise differences in Arlequin (Table 2). The ratio of pollen to seed ratio was low at 2.96 95% CI [2.69–3.23] (Table 2).

### 3.3. Molecular Dating and Evolutionary Relationships

#### 3.3.1. *Corymbia hamersleyana*

**Figure 5:**
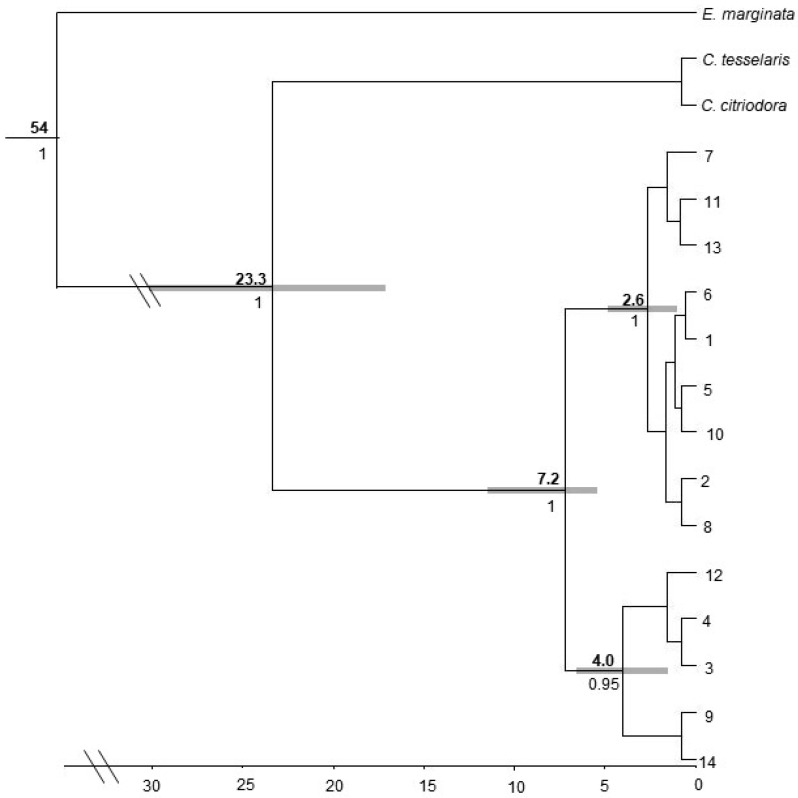
Phylogenetic tree of *C. hamersleyana* constructed from three cpDNA regions. Bold numbers above nodes indicate age in millions of years, numbers below lines are posterior probabilities of well supported internal nodes. Double slash lines indicate truncation of branch lengths for ease of visualisation. Note the tree shown for *C. hamersleyana* was dated based on the mean stem age of the *Corymbia* (*Angophora*) clade [65]. Grey boxes represent 95% confidence intervals (HPD). Scale bar represents time (Ma).

The maximum credibility tree from each of the three dating analyses generated using BEAST produced similar topologies showing two highly divergent clades, one consisting of five haplotypes and the other of nine. The three dating methods estimated these clades to have diverged between (a) 7.2 Ma (3.9–11.3 95% HPD), (b) 6.5 Ma (3.5–10.3 95% HPD), and (c) 5.5 Ma (3–8.5% HPD). Within the smaller clade all three differently calibrated trees indicated moderate support (>94% posterior probability) for further division into two sub-clades separating three haplotypes (Hap 3, 4, and 12) from Hap 9 and 14. Within the larger clade all three analyses indicated high support (100% posterior probability) for further division into two subclades separating three haplotypes (Hap 7, 11, and 13) from the remaining six (Hap 5, 2, 10, 8, 1, and 6) (Figure 5). Neither clade showed evidence of spatial clustering and haplotypes originating from the two most divergent clades were sampled from the same location in 11 of the 20 populations. This apparent lack of phylogeographic structure was supported with comparisons of *N*_ST_ with *G*_ST_, where *N*_ST_ was not significantly higher than *G*_ST_ (*N*_ST_ = 0.384, *G*_ST_ = 0.446, *p* > 0.05). The Median Joining network clearly delineates the two highly divergent clades, with the larger clade showing a star-like topology with the common and widespread Hap 1 in the centre (Figure 1). We did not observe that populations associated with the Chichester and Hamersley Ranges had higher haplotype diversity in comparison to the surrounding regions. 

#### 3.3.2. *Acacia pruinocarpa*

**Figure 6:**
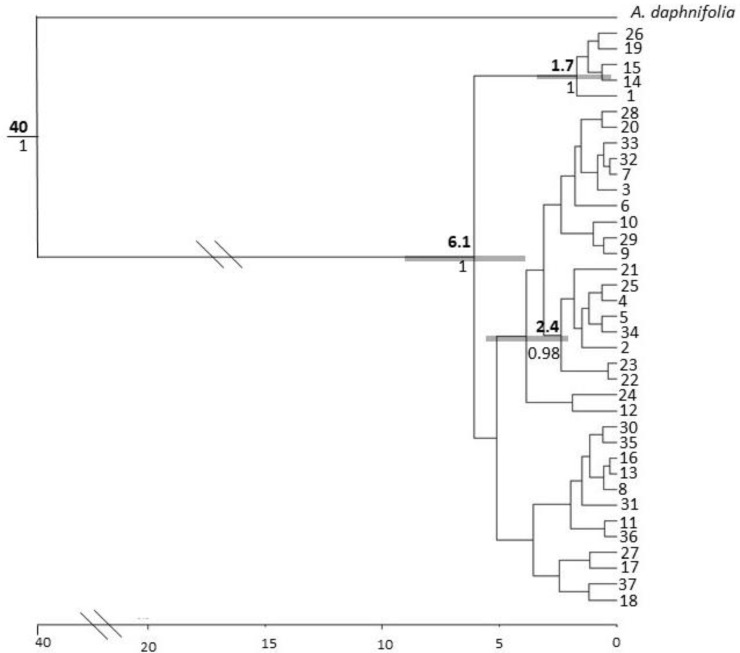
Phylogenetic tree of *A. pruinocarpa* constructed from four cpDNA regions. Numbers above nodes indicate age in millions of years, numbers below lines are posterior probabilities of well supported internal nodes. Double slash lines indicate truncation of branch lengths for ease of visualisation. Note the tree was dated based on the cpDNA substitution rate identified for the Mimosoid clade in [70]. Grey boxes represent 95% confidence intervals (HPD). Scale bar represents time (Ma).

The phylogenetic tree for *A. pruinocarpa* showed little resolution, except for the first node splitting five highly divergent haplotypes (Hap 1, 14, 15, 26, and 19) from all other haplotypes and a secondary internal node that split eight haplotypes (Hap 2, 4, 5, 21, 25, and 34). All other internal nodes were poorly supported (Figure 6). Based on the mean cpDNA mutation rate identified for the Mimosoid clade, the two major clades are estimated to have diverged approximately 6.1 Ma during the Miocene, but potentially as young as the Pliocene (3.2–9.9 Ma 95% HPD). Despite the poor node support for much of the MCC tree, there was still some evidence of phylogeographic structuring when comparing estimates of *N*_ST_ (0.489) with *G*_ST_ (0.366) with *p* < 0.01. The network of haplotype relationships reflects the complexity seen in the Bayesian tree with two areas of star-like phylogenetic patterning around the common haplotypes, Hap 4 and 8, and the presence of highly divergent haplotypes interspersed throughout the network (Figure 2). 

## 4. Discussion

Our study of two widespread Pilbara trees has shown patterns of haplotype diversity that are indicative of long-term persistence of these species within this landscape. However, we found no evidence of higher genetic diversity associated with topographic features such as the Hamersley and Chichester Ranges, and lower diversity in surrounding areas, as would be expected if these features acted as historical refugia. Some haplotypes were highly divergent indicating that historically at least; there has been isolation of some populations. The distribution of haplotype diversity indicated extensive gene flow, particularly via seed dispersal in both species and it appears that this seed movement has re-distributed genetic variation, such that highly divergent haplotypes now routinely co-occur within populations throughout the region. Genetic connectivity was also observed in the nuclear data, suggesting that contemporary gene flow via seed and pollen is high in both species. Below we examine how these processes of persistence and subsequent gene flow might have arisen throughout this landscape.

### 4.1. Historical Persistence

A key pattern that emerged from the cpDNA data in both tree species was the presence of many haplotypes. High diversity was also identified in two other widespread Pilbara tree/shrub species [20,21] and provides important insight into the evolutionary history of the landscape [72]. In populations of relatively equal size that are at, or approaching mutation-drift equilibrium, historical persistence is predicted to result in the accumulation of genetic variation [10,73,74] and our observations here indicate historical persistence in localised populations. This pattern has also been observed in flora from other ancient landscapes where extinction rates are low and topographic complexity and/or a lack of glaciation have preserved populations and their genetic diversity [75]. Thus, studies in regions such as the SWAFR [6,7,15], the GCFR [4], eastern North America [8], and the CA-FP [5] all demonstrate widespread tree species possessing cpDNA haplotype diversity far exceeding that typically encountered in European or eastern American species [5]. 

The high levels of divergence observed amongst several haplotypes in this study suggest that some of these populations have been historically isolated, allowing for drift of genetic lineages. Using only contemporary samples it is not possible to determine when this period of population isolation occurred. The phylogenetic trees show basal lineage diversification in both species to have occurred during the Miocene—an important period in the evolution of taxa within the Pilbara landscape [12]. Expansion of the Antarctic ice sheet and subsequent changes in precipitation during this time led to the aridification of central and north-western Australia, including the Pilbara, from the mid Miocene approximately 15 Ma [12,76,77,78]. It has been postulated that the topographical complexity of the Pilbara landscape provided refugia for both inter and intra-specific lineages during this aridification [12]. Persistence throughout aridification allowed for the generation of highly divergent lineages now seen in the endemic flora and fauna—many of which show coalescence during this time [76,79,80,81,82,83]. The timing of basal lineage diversification observed here is considerably older than that commonly reported for tree species from the SWAFR, including the congener of *C. hamersleyana*, *C. calophylla*, which have typically shown intra-specific haplotype differentiation to occur during the climatic fluctuations associated with the later Pliocene/Pleistocene period [16,17,84,85,86]. It is also older than the estimated lineage coalescence of trees originating from GCFR that showed diversification during the Pleistocene [4,87]. 

Although the timing of lineage diversification was similar for both tree species, the structuring of lineage diversification varied, with *C. hamersleyana* consisting of two, well-supported, highly divergent clades that further separate into well-supported lineages coalescing approx. 5.5–7.2 Ma. In contrast, *A. pruinocarpa*, showed diversification of approx. 6.1 Ma but few internal clades were well supported. The deep divergence observed in *C. hamersleyana* is striking given the absence of genetic structure in the nuclear DNA and because haplotypes from each of the divergent clades routinely co-occurred within populations. Discordance in cpDNA and nuclear gene trees as a result of introgressive hybridization is well documented amongst the eucalypts [88,89,90,91] and is increasingly being recognised in *Corymbia* species, where inter-sectional hybrids have been observed between distantly related taxa [92]. Indeed, non-monophyletic patterns of cpDNA variation have already been observed in *C. hamersleyana* [93] and appear to corroborate previous reports that hybridisation and introgression are a feature of this species [30]. While we found no nuclear structure that could be used to substantiate hybridisation, there are two possible explanations for the observed admixture of divergent clades. It may be due to historical unidirectional introgressive hybridisation that is no longer evident in the nuclear genome, with signatures of the process still visible in divergent haplotypes in the cpDNA. Alternatively, or in combination, there may be ongoing hybridisation with other Pilbara *Corymbia* species, and this would become apparent upon examination of nuclear patterns in sympatric species. Our sampling for this study has not allowed us to disentangle these processes, but we suggest that further exploration of the role of hybridisation in Pilbara bloodwoods would be informative. 

### 4.2. Topographic Refugia

One aspect of phylogeographic interpretation is to determine whether the distribution of genetic diversity reveals the presence of historical refugia within a landscape. Areas of high haplotype diversity and/or high nuclear diversity, contrasting with surrounding areas of shared low diversity, may indicate regions where species have persisted and/or contracted to during periods of unfavourable climate change with subsequent expansion when conditions were more suitable [74]. In the Pilbara tree species *Eucalyptus leucophloia*, the upland, topographically complex areas of the Hamersley and Chichester Ranges were identified as putative refugia [21] in the same way as for local fauna [82,83,94]. However, we found no evidence for these features acting as refugia for *C. hamersleyana* indicating that patterns may be largely idiosyncratic in unglaciated landscapes where biotic responses to changing climatic conditions are likely to be species specific. Due to limited sampling of lowland *A. pruinocarpa* populations we were unable to explicitly test the topographic refugia hypothesis, although the few samples available showed no differences with those from the Ranges. Further sampling, in particular to the south of the Pilbara is needed to confirm these patterns. 

### 4.3. Genetic Connectivity

The use of both nuclear and cpDNA markers in phylogeographic studies allows for distinction between processes of gene flow occurring via pollen and seed. Neutral, nuclear DNA variation reflects pollen and seed dispersal whereas cpDNA, when maternally inherited, reflects only seed movement [95]. Furthermore, different types of genetic markers provide insight into patterns of connectivity occurring at different timescales. Rapidly mutating microsatellite markers inform more recent patterns of pollen and seed flow whereas slowly evolving cpDNA markers are reflective of historical patterns of seed movement [96]. Patterns of nuclear DNA variation indicate that contemporary connectivity amongst populations of both *C. hamersleyana* and *A. pruinocarpa* is high. STRUCTURE analyses both showed high levels of admixture, and whilst this model has been shown to produce biased estimates under some conditions—such as isolation by distance or difficulty choosing the appropriate K value [97,98]—the results indicated a lack of genetic structure and a single genetic population in each species. Low levels of genetic structure in the microsatellite data were also identified under the infinite allele (*F*_ST_) and stepwise mutation (*R*_ST_) models suggesting there are currently few barriers to pollen and seed movement throughout the landscape, including distance, as peripheral and more isolated populations retained high levels of genetic diversity and low genetic divergence. These attributes indicate the maintenance of large effective population sizes over recent timescales [99,100]. High levels of genetic connectivity were also identified in two other Pilbara tree/shrub species with widespread distributions, the eucalypt *E. leucophloia* [21], and the wattle *Acacia ancistrocarpa* [20], although in contrast, limited connectivity was observed in two shrub species with restricted distributions in the Pilbara [20,22]. This pattern is consistent with theoretical expectations regarding the impact of widespread versus restricted distributions on gene flow and has been observed throughout a range of different regions and habitats [101,102]. High gene flow is often reported in widespread eucalypts [21] whereas widespread acacias have been associated with both extensive [20,103] and more restricted [104] gene flow. Interestingly, despite evidence of extensive genetic connectivity, all populations sampled (except the HAM population of *A. pruinocarpa*) showed significant *F*_IS_ values, which could indicate some level of inbreeding, including crossing between clonal ramets or due to structuring and crossing between related individuals within populations [104]. 

The cpDNA data that reflects historical gene flow processes were also informative. As discussed previously, the presence of highly divergent haplotypes in both species suggests that historically at least, populations of these trees have been isolated, allowing for the development of divergent haplotypes. The general lack of phylogeographic structure observed in this study, reflected in the presence of several common and widespread haplotypes as well as in the co-occurrence of highly divergent haplotypes, could arise via subsequent dispersal of these divergent lineages by seed or may indicate incomplete lineage sorting of ancestral polymorphisms. However, incomplete lineage sorting is more problematic when working at shallow time depths [105], and the patterns of deep divergence observed between haplotypes here, along with patterns seen in the nuclear data, point to seed flow as the cause of current phylogeographic patterns in the cpDNA and is suggestive of long-distance seed dispersal (LDD). This was confirmed with low ratios of pollen to seed dispersal in both species in comparison to the median pollen:seed ratio reported for a range of plant taxa [106]. Our results were also in stark contrast to other ratio estimates from closely related tree species; *Corymbia calophylla* from south-western Australia had a much higher pollen to seed ratio (68:1) [16] and the eucalypt, *Eucalyptus gomphocephala*, showed pollen migration rates up to 200 times higher than seed migration rates [107]. Other eucalypts have shown pollen migration rates to be 18 to 581 times higher than seed migration rates [7]. Interestingly, the two species studied here have quite differing seed morphology associated with dispersal as *C. hamersleyana* possesses winged seeds that may assist in wind dispersal while the small aril in *A. pruinocarpa* suggests ant dispersal [32]. 

Comparative phylogeographic analysis of some plant species has allowed direct observation of the impact of LDD on the formation of genetic structure. For example, in the Albany Subtropical Thicket Region of southern Africa, analysis of three widespread, sympatric trees was conducted to determine whether watersheds were barriers to seed dispersal [4]. All three species possessed multiple haplotypes and two species, one with wind-dispersed seed and one with bird-dispersed seed, showed strong phylogeographic structuring. In contrast, the third species did not show any structuring according to drainage systems, and patterns indicated that mammals, most likely elephants, were facilitating long-distance seed dispersal across watershed barriers [4]. The finding that four unrelated tree species of the Pilbara all experience extensive gene flow via the seed provides support for the role of landscape specific processes facilitating LDD. 

The presence of extreme meteorological events is considered to be a contributing factor in gene flow [108] and it has been proposed that this may be a significant factor in the extensive genetic connectivity observed in Pilbara plants [21]. The Pilbara is distinct from surrounding regions, and indeed many other parts of the world, in that it is subject to prolonged hot and dry periods followed by significant tropical depressions and intense cyclonic activity [29]. The intense wind gusts and surface water flows associated with these events have previously been hypothesised as major mechanisms of dispersal for plant species [109], including those from the Pilbara [20,21]. The cyclonic activity here occurs predominantly throughout December to March [29] and this is ideal timing for the dispersal of seeds, including those of *C. hamersleyana* and *A. pruinocarpa*, that experience fruit dehiscence during the summer months [30,32]. Dispersal via meteorological events such as flooding is particularly important given these forces can impact seeds of a range of taxa with varying morphology [108].

There are also a number of other features of the Pilbara that may contribute to gene flow and colonisation processes in the biota [108,110]. The first is the open nature of the terrestrial landscape where sparse and/or low vegetation allows the unfettered movement of seed and/or their vectors [108]. The Pilbara is characterised by open, low-lying vegetation that is dominated by species such as spinifex (*Triodia* spp.), ribbon grass (*Chrysopogon fallax*), and the scattered wattles such as kanji bush (*Acacia inaequilatera*), allowing ease of seed movement both via animal vectors and wind [111]. The second feature is the presence of large migratory animals, such as emus and kangaroos that provide opportunities for LDD via endozoochory or exozoochory [108,112]. Emus, for example, have previously been identified as vectors of LDD for a large variety of plant species. Their high mobility and long gut retention times allow for the transport of viable seeds that typically experience limited (e.g., ant-dispersed) or unassisted dispersal syndromes [112]. The now locally extinct environmental engineers—bandicoots, bettongs, and other small macropods as well as larger rodents would also have likely played key roles in seed dispersal, and whilst distances covered in single events may not have been large, over time the contribution may have been substantial. The third feature is the potential role of human transportation in the dispersal of seeds throughout this region. Indigenous Australians have occupied the Pilbara for at least 50 thousand years [113] and may have been important dispersers of some plant species [114]. Relatively little recognition has hitherto been given to the potential impacts of this means of dispersal on the present distributions of species and their genetic diversity, but it is likely that people have impacted the current biogeography of plant species, mobilising them for their use as food, medicine or for totemic purposes [114,115,116,117]. Combined with extreme meteorological events, these features provide a context for understanding the LDD observed in widespread Pilbara plant species and can form the basis of future hypotheses regarding genetic connectivity amongst the Pilbara flora. 

## 5. Conclusions

Our phylogeographic analysis has revealed important shared characteristics of two widespread tree species endemic to the Pilbara region, in particular, the ability of seed to travel long distances despite exhibiting different seed morphology associated with dispersal. In addition, the identification of a Miocene origin of divergent lineages in both species confirms the long-term persistence of these taxa in this ancient landscape and suggests that the intensifying aridity associated with this period was a critical driver of evolution in these Pilbara species. The findings of extensive genetic connectivity and a general lack of genetic structure are important given that seed sourcing for dryland restoration efforts is an ongoing activity within this region. 

## Figures and Tables

**Table 1:** Genetic diversity parameters for 20 populations of *C. hamersleyana* and 23 populations of *A. pruinocarpa* across the Pilbara region of north-western Australia. All parameters (except the total number of private alleles) are averaged across 14 nuclear microsatellite loci for each population. Standard errors are shown in parentheses. Presence on either Chichester or Hamersley Range is indicated.

Pop	*n*	*p*	*N*_A_	*N*_AP_	*H*_O_	*H*_E_	*F*_IS_	Range
***C. hamersleyana***								
BEN	24	85.71	5.57 (0.88)	1	0.47 (0.08)	0.58 (0.08)	0.18 *	
COP	24	100	6.11 (1.04)		0.47 (0.08)	0.58 (0.08)	0.19 *	
DAW	24	100	6.63 (1.14)		0.49 (0.07)	0.60 (0.08)	0.18 *	Chichester
DEE	24	100	6.52 (0.94)	1	0.52 (0.06)	0.63 (0.06)	0.17 *	
DIN	24	100	7.29 (1.04)	3	0.47 (0.07)	0.64 (0.06)	0.26 *	Hamersley
EMU	24	100	6.97 (1.04)	2	0.51 (0.08)	0.61 (0.08)	0.15 *	Hamersley
GAL	24	100	7.33 (1.24)		0.42 (0.07)	0.60 (0.09)	0.29 *	
GID	24	100	7.65 (1.25)		0.47 (0.07)	0.62 (0.08)	0.23 *	Hamersley
HAM	24	100	7.06 (1.26)		0.52 (0.07)	0.61 (0.08)	0.14 *	Hamersley
HOO	24	100	6.61 (1.11)		0.49 (0.08)	0.57 (0.08)	0.14 *	Chichester
KAN	24	100	6.84 (1.02)		0.46 (0.07)	0.59 (0.07)	0.21 *	
KAR	24	100	7.00 (1.17)	2	0.47 (0.07)	0.60 (0.08)	0.21 *	
MCK	24	100	6.22 (1.10)	3	0.49 (0.07)	0.58 (0.07)	0.15 *	Chichester
MIN	24	100	6.54 (0.84)	2	0.49 (0.08)	0.59 (0.07)	0.17 *	
NUL	24	100	6.21 (0.91)	1	0.46 (0.08)	0.62 (0.08)	0.25 *	
ORD	24	92.86	6.68 (1.08)		0.50 (0.07)	0.62 (0.08)	0.19 *	
PAM	24	92.86	7.08 (1.16)	1	0.47 (0.08)	0.59 (0.09)	0.20 *	Hamersley
RUB	24	92.86	6.40 (1.15)	3	0.52 (0.08)	0.59 (0.08)	0.12 *	
SHA	24	100	6.30 (0.98)	1	0.51 (0.06)	0.64 (0.07)	0.19 *	
WEE	24	100	6.44 (1.05)	1	0.45 (0.08)	0.60 (0.08)	0.24 *	Hamersley
Mean (SE)	24.00 (0)	98.21 (0.88)	6.67 (0.11)	1.88 (0.27)	0.48 (0.01)	0.60 (0.01)	0.19 (0.01)	
***A. pruinocarpa***								
ANG	20	92.86	4.69 (0.71)		0.42 (0.07)	0.52 (0.07)	0.20 *	Hamersley
BUN	24	100	4.55 (0.70)		0.48 (0.05)	0.58 (0.06)	0.18 *	
COO	23	92.86	4.68 (0.71)	1	0.41 (0.06)	0.57 (0.07)	0.29 *	
COR	24	85.71	4.01 (0.66)	2	0.46 (0.07)	0.50 (0.07)	0.08 *	Chichester
HAM	24	92.86	4.33 (0.78)	1	0.54 (0.07)	0.55 (0.07)	0.02	Hamersley
HAR	24	100	4.41 (0.73)	1	0.45 (0.07)	0.50 (0.07)	0.11 *	Hamersley
HES	23	92.86	4.86 (0.85)	1	0.41 (0.06)	0.51 (0.07)	0.20 *	Chichester
HIL	24	92.86	5.05 (0.91)	1	0.45 (0.06)	0.54 (0.07)	0.18 *	Hamersley
HRP	24	85.71	4.57 (0.95)		0.40 (0.08)	0.47 (0.08)	0.15 *	Chichester
MSJ	23	92.86	4.49 (0.80)	1	0.45 (0.07)	0.54 (0.07)	0.18 *	
MET	22	92.86	4.90 (0.94)		0.47 (0.07)	0.56 (0.08)	0.16 *	Hamersley
NAM	24	92.86	4.45 (0.78)	1	0.42 (0.07)	0.50 (0.08)	0.17 *	Hamersley
OPH	23	100	4.66 (0.64)	3	0.41 (0.07)	0.53 (0.07)	0.24 *	Hamersley
OXE	23	100	4.67 (0.77)		0.43 (0.06)	0.54 (0.07)	0.20 *	Hamersley
RHL	20	92.86	4.06 (0.63)		0.41 (0.07)	0.50 (0.08)	0.18 *	Chichester
RHO	23	92.86	3.87 (0.56)		0.39 (0.07)	0.46 (0.08)	0.14 *	Hamersley
RIO	23	92.86	4.25 (0.78)	1	0.43 (0.07)	0.51 (0.07)	0.17 *	Hamersley
SPE	23	92.86	4.58 (0.90)	1	0.42 (0.07)	0.51 (0.07)	0.17 *	Hamersley
STE	24	100	4.18 (0.69)		0.35 (0.07)	0.47 (0.07)	0.25 *	
WAR	24	100	4.56 (0.66)	1	0.46 (0.07)	0.55 (0.07)	0.15 *	Hamersley
WRA	24	92.86	4.80 (0.84)	1	0.44 (0.08)	0.47 (0.07)	0.05 *	Hamersley
YAM	24	85.71	4.58 (0.83)		0.42 (0.07)	0.49 (0.08)	0.14 *	Hamersley
YAR	24	100	4.59 (0.76)		0.42 (0.06)	0.53 (0.07)	0.21 *	Hamersley
Mean (SE)	23.22(0.24)	94.10 (0.97)	4.51 (0.06)	1.23 (0.17)	0.43 (0.01)	0.52 (0.01)	0.17 (0.01)	

*n*: Number of individuals; *p*: Percent polymorphic loci; *N*_A_: Allelic richness; *N*_AP_: Total number of private alleles; *H*_O_: Observed heterozygosity; *H*_E_: Expected heterozygosity; *F*_IS_: Fixation index. * Highly significant *p* < 0.005.

**Table 2:** Chloroplast DNA diversity statistics, tests for neutrality, and demographic and spatial expansions based on three sequenced regions (rpl16 intron, ndhC-trnV, trnG intron) from 20 populations of *C. hamersleyana*, and four sequenced regions (ndhF-rpl32, rpl32-trnL, trnS-trnG, psbD-trnT) from 23 populations of *A. pruinocarpa*.

Species	*h*	*hd*	π	Tajima’s D	Fu’s *F*_s_	*R*_2_	Demographic Expansion	Spatial Expansion	Pollen/Seed Flow Ratio
*C. hamersleyana*	14	0.759	0.003 (0.00)	0.607 ns	2.474 ns	0.086 *	SSD (*p* = 0.31):Hrag ^NS^	SSD (*p* = 0.51):Hrag ^NS^	6.67 (6.45–6.89)
*A. pruinocarpa*	37	0.903	0.002 (0.00)	−0.844 ns	−4.982 ns	0.082 *	SSD (*p* = 0.42):Hrag ^NS^	SSD (*p* = 0.49):Hrag ^NS^	2.96 (2.69–3.23

Number of haplotypes (*h*), haplotype diversity (*hd*), nucleotide diversity (π). Estimates of pollen to seed flow ratios (95% confidence intervals in parentheses) (Ennos, 1994). * Highly significant *p* < 0.005.

## Supplementary Materials

The following are available online at https://www.mdpi.com/2073-4425/11/8/863/s1. Table S1: Details for the populations of *Corymbia hamersleyana* (*n* = 20) sampled across the Pilbara region of north-western Australia; Table S2: Details for the populations of *Acacia pruinocarpa* (*n* = 23) sampled across the Pilbara region of north-western Australia; Table S3: Primer sequences and characteristics of microsatellite loci developed for *Corymbia hamersleyana* (CH) and *Acacia pruinocarpa* (AP); Table S4: Estimates of global *F*_ST_ of Weir (1996) both using and without using the ENA correction described in Chapuis and Estoup (2007); Figure S1: Results of STRUCTURE analyses of *C. hamersleyana* showing Delta *K* vs. *K* and bar plots of individual assignment to clusters *K* = 2, 6, 8, 13, and 15; Figure S2: Results of STRUCTURE analysis of *A. pruinocarpa* showing Delta *K* vs. *K* and bar plots of individual assignment to clusters *K* = 2, 6, 8, 13, and 15; Figure S3: Principal co-ordinates analysis based on microsatellite data for individuals of A) *Corymbia hamersleyana* and B) *Acacia pruinocarpa* sampled across the Pilbara region of Western Australia.
