# Supplementary material for: Extensive Genetic Connectivity and Historical Persistence Are Features of Two Widespread Tree Species in the Ancient Pilbara Region of Western Australia
Source: Genes (Basel). 2020 Jul 29;11(8):863. doi: 10.3390/genes11080863 (PMC7465080; doi:10.3390/genes11080863)

Supplementary material

Table S1. Details for the populations of *Corymbia hamersleyana* (n=20) sampled across the Pilbara region of north-western Australia. Haplotype ID, Haplotype diversity and Genbank accession numbers shown. Sequence alignment accession: 10.6084/m9.figshare.11846940

Location	Pop.	Latitude	Longitude	Hap #	Hap. diversity	Genbank accession rpl16	ndhC-trnV	trnG
Bennett	BEN	22° 57' 33.7" S	117° 51' 52.2" E	1,2	0.571	MN539542	MN539548	MN539557;MN539558
Coppin	COP	20° 55' 17.5" S	119° 58' 49.8" E	3,4	0.25	MN539543	MN539549	MN539559;MN539560
Dawson	DAW	21° 34' 34.3" S	117° 12' 47.0" E	3,5	0.536	MN539542:MN539543	MN539549;MN539550	MN539557;MN539559
Deepdale	DEE	21° 38' 33.0" S	116° 05' 18.2" E	6,7	0.429	MN539542;MN539543;MN539544	MN539549;MN539551;MN539552	MN539557
Dinner	DIN	22° 34' 32.3" S	118° 18' 03.3" E	1,3	0.25	MN539542	MN539548	MN539557;MN539559
East Munjina	EMU	22° 29' 22.5" S	118° 44' 10.3" E	1,3	0.25	MN539542;MN539543	MN539548;MN539549	MN539557;MN539559
Gallop	GAL	21° 26' 53.4" S	120° 04' 24.8" E	1,3	0.25	MN539542;MN539543	MN539548;MN539549	MN539557;MN539559
Gidge	GID	22° 29' 19.8" S	119° 01' 09.5" E	1,3,5	0.679	MN539542;MN539543	MN539548;MN539549;MN539550	MN539557;MN539559
Hamersley	HAM	22° 15' 10.5" S	117° 40' 24.7" E	1,3,8	0.679	MN539542;MN539543	MN539548;MN539549;MN539553	MN539557;MN539559
Hooley	HOO	21° 49' 58.3" S	117° 55' 39.6" E	1,3	0.429	MN539542;MN539543;MN539545	MN539548;MN539549	MN539557;MN539559
Kangan	KAN	21° 02' 06.2" S	118° 39' 45.3" E	1,7,9	0.607	MN539542;MN539546	MN539548;MN539552;MN539554	MN539557;MN539561
Karratha	KAR	20° 44' 34.6" S	116° 52' 19.8" E	10	0.25	MN539542	MN539548	MN539557;MN539562
McKay	MCK	22° 25' 16.9" S	119° 59' 29.4" E	1,3,11	0.607	MN539542;MN539543;MN539545	MN539548;MN539549;MN539555	MN539557;MN539559
Minnie	MIN	21° 55' 48.9" S	115° 16' 23.1" E	1	0	MN539542	MN539548;MN539551	MN539557
Nullagine	NUL	21° 53' 19.6" S	120° 05' 55.6" E	1,3,11	0.607	MN539542;MN539543	MN539548;MN539549;MN539555	MN539557;MN539559
Ord	ORD	20° 19' 36.2" S	119° 10' 36.2" E	1	0	MN539542	MN539548	MN539557
Pamelia	PAM	23° 09' 24.7" S	119° 20' 55.5" E	1,3	0.429	MN539542;MN539543	MN539548;MN539549	MN539557;MN539559
Rubin	RUB	20° 23' 58.1" S	119° 58' 45.5" E	3,12	0.536	MN539543;MN539546	MN539549	MN539557
Shaw	SHA	20° 42' 35.1" S	119° 19' 06.2" E	5,9,13, 14	0.75	MN539542;MN539546;MN539547	MN539550;MN539554;MN539555;MN539556	MN539557;MN539561
Weeli Wolli	WEE	22° 55' 32.6" S	119° 11' 49.7" E	1,2,3	0.464	MN539542;MN539543	MN539548;MN539549	MN539557;MN539558

Table S2. Details for the populations of *Acacia pruinocarpa* (*n*= 23) sampled across the Pilbara region of north-western Australia. Haplotype number, Haplotype diversity and Genbank accession numbers shown. Sequence alignment accession: 10.6084/m9.figshare.11846937

**Location**	**Pop.**	**Latitude**	**Longitude**	**Hap #**	**Hap. diversity**	**Genbank accessionndhF-rpl32**	**rpl32-trnL**	**trnS-trnG**	**psbD-trnT**
Angelas	ANG	23° 05’ 05.9” S	118° 41’ 31.1” E	1,2,3,4	0.821	MN539563	MN539575;MN539576; MN539577	MN539585;MN539586; MN539587;MN539588	MN539603
Bungaroo	BUN	21° 51’ 52.9” S	116° 26’ 18.7” E	4,5,6,7	0.786	MN539563;MN539564	MN539576;MN539577	MN539588;MN539589	MN539603
Coondiner	COO	22° 42’ 46.6” S	119° 41’ 03.9” E	8,9,10	0.714	MN539563;MN539565	MN539577;MN539578	MN539588;MN539590	MN539603;MN539604
Corbay	COR	21° 57’ 56.1” S	118° 03’ 05.9” E	8,11,12	0.607	MN539563;MN539566; MN539567	MN539577;MN539578	MN539590;MN539591	MN539603;MN539604
Hamersley	HAM	22° 15’ 10.5” S	117° 40’ 24.7” E	4,8,13	0.571	MN539563	MN539576;MN539578; MN539579	MN539588;MN539590; MN539592	MN539603
Hardy	HAR	22° 57’ 14.2” S	117° 18’ 39.6” E	4	0	MN539563	MN539576	MN539588	MN539603
Harper	HRP	21° 52’ 16.3” S	117° 37’ 15.5” E	8	0	MN539563	MN539578	MN539590	MN539603
Hesta	HES	22° 13’ 31.7” S	119° 00’ 53.6” E	8,14,15,16	0.714	MN539563;MN539568; MN539569	MN539578;MN539580; MN539581	MN539590;MN539593	MN539603
Hillditch	HIL	23° 13’ 41.3” S	118° 45’ 32.6” E	7,9,17,18	0.821	MN539563;MN539570	MN539577	MN539588;MN539590; MN539594	MN539603;MN539604
Mesa G	MSJ	21° 39’ 10.2” S	116° 08’ 07.5” E	22,23	0.429	MN539563;MN539571	MN539576;MN539584	MN539588;MN539595	MN539605
Metawandy	MET	22° 40’ 10.3” S	116° 36’ 07.9” E	7,18,19,20,21	0.857	MN539563	MN539577;MN539581; MN539582;MN539583	MN539588;MN539590; MN539594	MN539603
Nameless	NAM	22° 43’ 38.7” S	117° 44’ 57.5” E	3,24,25	0.679	MN539563	MN539576;MN539577	MN539587;MN539588; MN539596	MN539606;MN539607
Ophthalmia	OPH	23° 20’ 33.2” S	119° 49’ 51.4” E	8,10,26,27	0.643	MN539563;MN539565	MN539577;MN539578; MN539581;MN539582	MN539588;MN539590; MN539592;MN539594	MN539604
Oxer	OXE	22° 34’ 33.3” S	118° 13’ 43.1” E	3,4,28,8	0.607	MN539563	MN539576;MN539577; MN539578	MN539587;MN539588; MN539590;MN539597	MN539603
Rhodes	RHO	23° 03’ 18.4” S	119° 15’ 30.2” E	7,31	0.571	MN539563;MN539572	MN539577;MN539578	MN539588;MN539590	MN539603
Rio	RIO	22° 11’ 38.6” S	117° 57’ 19.9” E	4,18	0.536	MN539563;MN539573	MN539576;MN539577	MN539588;MN539590	MN539603
Roy Hill	RHL	22° 23’ 52.1” S	119° 59’ 27.9” E	8,9,29,30	0.786	MN539563	MN539577;MN539578	MN539588;MN539590; MN539598;MN539599	MN539603;MN539604
Spearhole	SPE	23° 21’ 30.9” S	119° 06’ 41.2” E	3,18	0.571	MN539563	MN539577	MN539587;MN539600	MN539603
Stewart	STE	21° 57’ 16.1” S	119° 33’ 36.5” E	8	0	MN539563	MN539578	MN539590	MN539603
Warrawanda	WAR	23° 43’ 56.9” S	119° 43’ 35.7” E	10,18,32,33	0.714	MN539563;MN539565; MN539569	MN539577	MN539587;MN539588; MN539590;MN539600	MN539603
Western Range	WRA	23° 10’ 19.1” S	117° 26’ 06.7” E	4,34	0.714	MN539563;MN539574	MN539576	MN539588	MN539603

Yampire	YAM	22° 27’ 18.6” S	118° 27’ 22.1” E	13,35,36	0.429	MN539563;MN539566	MN539578;MN539579	MN539590;MN539601	MN539603
Yarrintree	YAR	22° 36’ 49.8” S	118° 42’ 18.7” E	8,9,18,37	0.75	MN539563	MN539577;MN539578	MN539590;MN539595; MN539602	MN539603

Table S3. Primer sequences and characteristics of microsatellite loci developed for *Corymbia hamersleyana* (CH) and *Acacia pruinocarpa* (AP). Loci were used to genotype 24 individuals from 20 populations. Values indicate population mean. N, number of individuals analysed, N_A, allelic richness, H_O, observed heterozygosity, H_E, expected heterozygosity, F_{IS}, inbreeding coefficient. Annealing temperature for all loci was 60°C . Genotype table accession C.hamersleyana: 10.6084/m9.figshare.11846904; A. pruinocarpa: 10.6084/m9.figshare.11846952

Locus	Primer Sequence (5'-3')	Repeat motif	Size (bp)	N	N_A	H_O	H_E	F_{IS}
CH1	F: TGTTCGCAAGAGCTTCATGT<br>R: TTGCTAATGTCCCAAGGAGAA	$(AG)_5$	82-98	23.45	3.3	0.26	0.269	0.033
CH6	F: CTGGTCCGAGACTGAAGCAT<br>R: ATTTCTCCACGAACCCGAC	$(AG)_7$	106-118	24	2.45	0.096	0.118	0.186
CH9	F: AAGACCCATCTCCACAGTCG<br>R: GGAGCACACCCAACATCTCT	$(AG)_7$	128-198	23.05	13.15	0.636	0.856	0.257
CH10	F: GCCATGCTTATTCGGCTTT<br>R: CCGACGTCTTCGTCTTCACT	$(AG)_6$	130-136	23.8	2.95	0.372	0.395	0.057
CH11	F: TGCTCGATCATCCGACTATG<br>R: TTAGGGCTTTCCAGAGCAAA	$(AG)_9$	130-177	23	12.05	0.685	0.866	0.209
CH14	F: AAGGTCAAGCATGGACTTTCA<br>R: ATTCACGAGTTCCCATCTGC	$(AC)_5$	147-153	24	2	0.121	0.128	0.059
CH17	F: TCGTTGCCGACATAAGTCCT<br>R: AAACGCTCCGAGCTCTTTCT	$(AG)_7$	149-167	23.95	3.55	0.474	0.487	0.027
CH18	F: AATCTTGAGCATCGCCAATC<br>R: CAGGAATCTCGCCATCAACT	$(AGG)_7$	161-185	24	5.65	0.752	0.682	-0.103
CH22	F: GTTATACCGTTCACGCCACC<br>R: CAGCCAGGTCTCGAAGTAGG	$(AG)_{10}$	181-227	23.75	12.7	0.818	0.854	0.041
CH35	F: GCGTCGAAGAAGATGACGAT<br>R: CGGTAGCACTGCTTTCCAA	$(ACG)_8$	258-288	23.75	4.65	0.467	0.531	0.122
CH39	F: ATCGTGGTCGCTCTTCATCT<br>R: TTGTCTCCGCCCATGATAA	$(AG)_{12}$	100-130	23.4	9.45	0.704	0.827	0.148

CH42	F: ATGTCATCAACGCGTCTTGT<br>R: TCCAGTATCCAGATTATGCGG	$(AC)_8$	135-157	19.45	5.5	0.199	0.644	0.691
CH47	F: TTGTGCCTCTGTTTGCTGTT<br>R: CCTTGAATTCAGAGCTTGGG	$(AG)_{11}$	191-229	22.25	8.3	0.356	0.785	0.546
CH51	F: GCCGAATCCTAGGACAGAGA<br>R: TGTATCCAAACATCGAAAGGC	$(AG)_9$	299-343	23.6	10.45	0.809	0.823	0.016
AP05	F: GCTTCTCCTGTTGCTTGTCC<br>R: TTGTGGACCTCTTGGTCTGG	$(AAC)_6$	106-114	23.22	1.7	0.069	0.068	-0.016
AP11	F: TGTTGTCCATACCTGAGGGC<br>R: GGTCCGCTTTCCCATTCTAT	$(AG)_9$	115-144	23.22	4.87	0.428	0.659	0.351
AP14	F: TGGATCAATCCTGTTACCCA<br>R: GCATCCACTTGGACAGATCC	$(AAG)_6$	134-149	23.22	2.96	0.518	0.498	-0.04
AP15	F: GGCAAGCAACTCTTACCGAA<br>R: ACATTCCGTGAATCGAAAGG	$(ACT)_6$	144-150	23.22	2.04	0.068	0.452	0.207
AP21	F: GCGATGAATATCGTCGAACA<br>R: CCCTTCCTTTGCCCAACTAT	$(AG)_{10}$	171-201	23.13	9.17	0.844	0.823	-0.025
AP37	F: CTTCTCCCATTCTTTGCTCG<br>R: TTACTATGCCCTCCTGCCTG	$(AAG)_6$	86-107	22.04	3.43	0.417	0.416	-0.003
AP38	F: GCTTATCTTTGATGCAGGGC<br>R: TTGGTGGACCCACTGATGT	$(AG)_{11}$	93-117	23.13	6.78	0.608	0.741	0.18
AP39	F: GATTCACTGTCCGGCCTCT<br>R: AGTATAACGCCGTTGGCAAT	$(AG)_8$	104-110	23.22	2.7	0.252	0.249	-0.012
AP43	F: AAATGCGATGTTGGATGAGG<br>R: TTGACAAGTCCAAGGAACCC	$(AAG)_6$	122-140	22.96	3.57	0.314	0.554	0.433
AP44	F: GAACCCACAAGGTTCTCCAA<br>R: CCAACACGTAGGGATGGAGT	$(AGG)_6$	131-134	23.22	1.7	0.089	0.093	0.049
AP48	F: GCTTGGTATCAACGCAGATG<br>R: CTCCCAACAAGTGCTCTTCC	$(AAT)_{13}$	138-190	21.43	8.78	0.461	0.798	0.422
AP52	F: GGTCTGTCGTTGTTGCTGAG<br>R: ACCCAATATCGGAAACACCA	$(AC)_7$	190-200	23.22	3.87	0.538	0.548	0.018

AP56	F: CCGACCGAAACTTGAGCTAC<br>R: CGGTGGCCACTATCACTGTA	$(AC)_{16}$	196-232	23.22	9.96	0.776	0.818	0.051
AP60	F: GCTTTCGACAGTTCTCAGGA<br>R: CTCCCAAGGAAGAACAACCA	$(AG)_{7}$	268-278	23.22	3.39	0.375	0.373	-0.006

Table S4. Estimates of global F_{ST} of Weir (1996) both using and without using the ENA correction described in Chapuis and Estoup (2007).

	Corymbia	*hamersleyana*	*Acacia*	*pruinocarpa*
locus	FST without ENA	FST with ENA	FST without ENA	FST with ENA
1	0.024953	0.033828	0.068962	0.080862
2	0.037304	0.034806	0.051368	0.052736
3	0.063402	0.063841	0.053933	0.056890
4	0.039019	0.035099	0.029996	0.042152
5	0.039403	0.038888	0.076366	0.074141
6	0.038115	0.035858	0.064094	0.063249
7	0.043063	0.058053	0.086067	0.074754
8	0.005898	0.035318	0.050109	0.049813
9	0.053859	0.052749	0.076608	0.077406
10	0.113549	0.108202	0.053923	0.049598
11	0.032972	0.033751	0.062431	0.062763
12	0.061974	0.049563	0.070483	0.069669
13	0.047541	0.037192	0.039838	0.039787
14	0.05375	0.051641	0.041530	0.042595
all loci	0.049884	0.047828	0.060318	0.058601
95% lower CI	0.040322	0.039145	0.051635	0.051644
95% upper CI	0.062223	0.059460	0.068624	0.065321

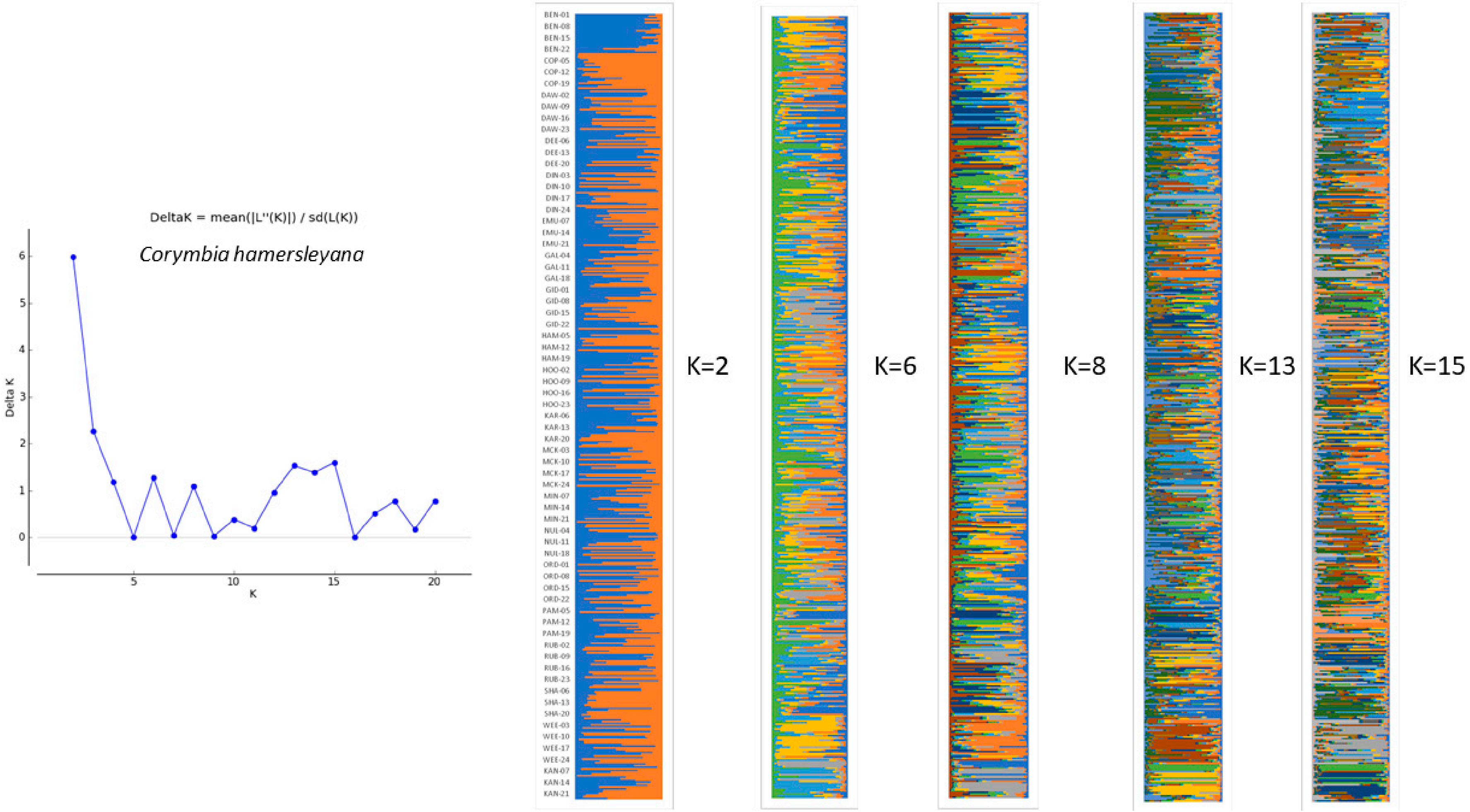

Fig. S1. Results of STRUCTURE analyses of *C. hamersleyana* showing Delta K vs K and bar plots of individual assignment to clusters K=2, 6, 8, 13 and 15.

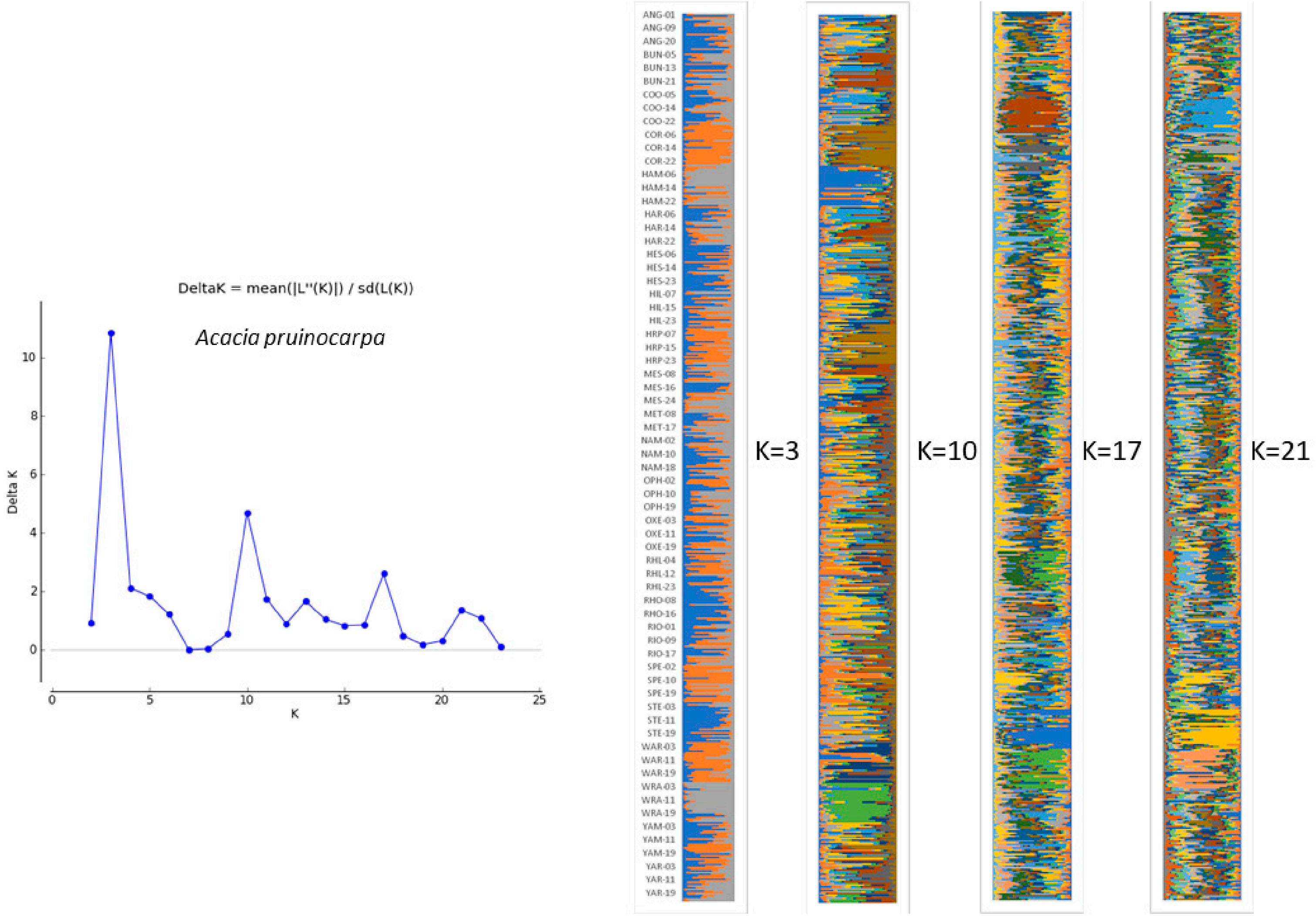

Fig. S2. Results of STRUCTURE analysis of *A. pruinocarpa* showing Delta K vs K and bar plots of individual assignment to clusters K=2, 6, 8, 13 and 15.

Fig. S3. Principal Co-ordinates Analysis based on microsatellite data for individuals of A) *Corymbia hamersleyana* and B) *Acacia pruinocarpa* sampled across the Pilbara region of Western Australia.

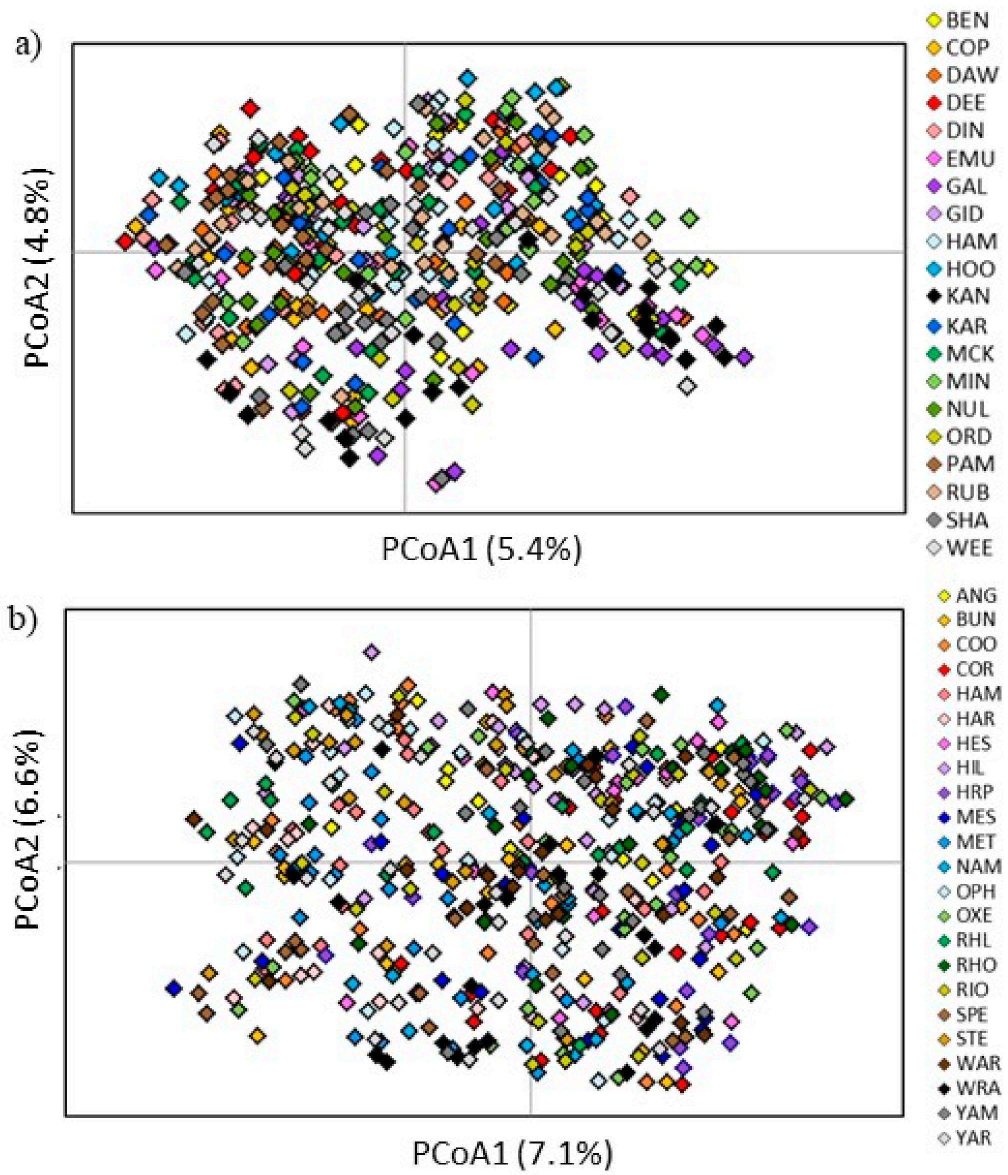

Supplement: Supplementary file 1 [file genes-11-00863-s001.pdf]
